# Supplementary material for: Transcriptome Analysis Reveals Vimentin-Induced Disruption of Cell–Cell Associations Augments Breast Cancer Cell Migration
Source: Cells. 2022 Dec 13;11(24):4035. doi: 10.3390/cells11244035 (PMC9776984; doi:10.3390/cells11244035)
Supplement: Supplementary file 1 [file cells-11-04035-s001.zip › DEGs and enrichment analysis.zip!/DEGs and enrichment analysis/WT_VIMvsVector KEGG/GSEA/WT_VIM/BASE_EXCISION_REPAIR/BASE_EXCISION_REPAIR(HSA03410).html]

Details for gene set BASE\_EXCISION\_REPAIR(HSA03410)[GSEA]

|  || Dataset | fpkm.sample |
| Phenotype | sample.cls |
| Upregulated in class | WT\_VIM |
| GeneSet | BASE\_EXCISION\_REPAIR(HSA03410) |
| Enrichment Score (ES) | 0.70194024 |
| Normalized Enrichment Score (NES) | 1.2400671 |
| Nominal p-value | 0.0 |
| FDR q-value | 0.26699984 |
| FWER p-Value | 0.335 |
Table: GSEA Results Summary

  

Fig 1: Enrichment plot: BASE\_EXCISION\_REPAIR(HSA03410)      
 Profile of the Running ES Score & Positions of GeneSet Members on the Rank Ordered List

  

| PROBE | DESCRIPTION (from dataset) | GENE SYMBOL | GENE\_TITLE | RANK IN GENE LIST | RANK METRIC SCORE | RUNNING ES | CORE ENRICHMENT || 1 | ENSG00000253463 | HMGB1P19 |  |  | 770 | Infinity | -0.0129 | Yes |
| 2 | ENSG00000248909 | HMGB1P21 |  |  | 1800 | Infinity | -0.0302 | Yes |
| 3 | ENSG00000213707 | HMGB1P10 |  |  | 3147 | 4.374 | 0.0008 | Yes |
| 4 | ENSG00000065057 | NTHL1 |  |  | 3149 | 4.346 | 0.0543 | Yes |
| 5 | ENSG00000062822 | POLD1 |  |  | 3291 | 3.989 | 0.1010 | Yes |
| 6 | ENSG00000168496 | FEN1 |  |  | 3538 | 3.632 | 0.1416 | Yes |
| 7 | ENSG00000106628 | POLD2 |  |  | 3557 | 3.585 | 0.1854 | Yes |
| 8 | ENSG00000270343 | UNGP3 |  |  | 3940 | 3.088 | 0.2169 | Yes |
| 9 | ENSG00000105486 | LIG1 |  |  | 4197 | 2.863 | 0.2478 | Yes |
| 10 | ENSG00000109674 | NEIL3 |  |  | 4599 | 2.616 | 0.2733 | Yes |
| 11 | ENSG00000132646 | PCNA |  |  | 4836 | 2.473 | 0.2997 | Yes |
| 12 | ENSG00000177084 | POLE |  |  | 5060 | 2.371 | 0.3251 | Yes |
| 13 | ENSG00000132967 | HMGB1P5 |  |  | 5689 | 2.141 | 0.3408 | Yes |
| 14 | ENSG00000100479 | POLE2 |  |  | 5908 | 2.060 | 0.3625 | Yes |
| 15 | ENSG00000114026 | OGG1 |  |  | 6063 | 2.014 | 0.3847 | Yes |
| 16 | ENSG00000076248 | UNG |  |  | 6695 | 1.879 | 0.3971 | Yes |
| 17 | ENSG00000132781 | MUTYH |  |  | 6856 | 1.852 | 0.4172 | Yes |
| 18 | novel.303 | - |  |  | 7402 | 1.735 | 0.4294 | Yes |
| 19 | ENSG00000255725 | TDGP1 |  |  | 7727 | 1.671 | 0.4444 | Yes |
| 20 | ENSG00000140398 | NEIL1 |  |  | 7743 | 1.668 | 0.4647 | Yes |
| 21 | ENSG00000005156 | LIG3 |  |  | 7834 | 1.651 | 0.4835 | Yes |
| 22 | ENSG00000103152 | MPG |  |  | 8232 | 1.588 | 0.4963 | Yes |
| 23 | ENSG00000269534 | AC011466.3 |  |  | 8453 | 1.552 | 0.5117 | Yes |
| 24 | ENSG00000169188 | APEX2 |  |  | 8544 | 1.542 | 0.5292 | Yes |
| 25 | ENSG00000259781 | HMGB1P6 |  |  | 8934 | 1.490 | 0.5410 | Yes |
| 26 | ENSG00000143799 | PARP1 |  |  | 8977 | 1.486 | 0.5585 | Yes |
| 27 | ENSG00000148229 | POLE3 |  |  | 9318 | 1.440 | 0.5705 | Yes |
| 28 | ENSG00000189403 | HMGB1 |  |  | 9388 | 1.432 | 0.5870 | Yes |
| 29 | ENSG00000154328 | NEIL2 |  |  | 9447 | 1.424 | 0.6035 | Yes |
| 30 | ENSG00000166171 | DPCD |  |  | 9583 | 1.407 | 0.6185 | Yes |
| 31 | ENSG00000115350 | POLE4 |  |  | 9648 | 1.399 | 0.6347 | Yes |
| 32 | ENSG00000077514 | POLD3 |  |  | 9747 | 1.389 | 0.6501 | Yes |
| 33 | ENSG00000073050 | XRCC1 |  |  | 10603 | 1.305 | 0.6517 | Yes |
| 34 | ENSG00000123415 | SMUG1 |  |  | 10881 | 1.276 | 0.6627 | Yes |
| 35 | ENSG00000041880 | PARP3 |  |  | 10941 | 1.272 | 0.6774 | Yes |
| 36 | ENSG00000166169 | POLL |  |  | 11044 | 1.260 | 0.6912 | Yes |
| 37 | ENSG00000248196 | AC092658.1 |  |  | 11308 | 1.236 | 0.7019 | Yes |
| 38 | ENSG00000100823 | APEX1 |  |  | 13004 | 1.102 | 0.6868 | No |
| 39 | ENSG00000124097 | HMGB1P1 |  |  | 14967 | 0.972 | 0.6655 | No |
| 40 | ENSG00000139372 | TDG |  |  | 15014 | 0.969 | 0.6767 | No |
| 41 | ENSG00000129484 | PARP2 |  |  | 15459 | 0.942 | 0.6808 | No |
| 42 | ENSG00000129071 | MBD4 |  |  | 17019 | 0.856 | 0.6649 | No |
| 43 | ENSG00000175482 | POLD4 |  |  | 17979 | 0.800 | 0.6585 | No |
| 44 | ENSG00000070501 | POLB |  |  | 19062 | 0.738 | 0.6493 | No |
| 45 | ENSG00000273628 | AL354798.1 |  |  | 21001 | 0.636 | 0.6243 | No |
| 46 | ENSG00000102699 | PARP4 |  |  | 22586 | 0.538 | 0.6041 | No |
| 47 | ENSG00000180189 | HMGB1P14 |  |  | 24526 | 0.397 | 0.5761 | No |
| 48 | ENSG00000253516 | HMGB1P41 |  |  | 25165 | 0.332 | 0.5694 | No |
| 49 | ENSG00000224976 | PARP4P2 |  |  | 27890 | 0.000 | 0.5232 | No |
| 50 | ENSG00000230611 | HMGB1P27 |  |  | 28011 | 0.000 | 0.5212 | No |
| 51 | ENSG00000259648 | AL132640.2 |  |  | 28935 | 0.000 | 0.5056 | No |
| 52 | ENSG00000270986 | HMGB1P51 |  |  | 29201 | 0.000 | 0.5011 | No |
| 53 | ENSG00000219163 | HMGB1P20 |  |  | 29243 | 0.000 | 0.5004 | No |
| 54 | ENSG00000224159 | HMGB1P9 |  |  | 29445 | 0.000 | 0.4970 | No |
| 55 | ENSG00000230519 | HMGB1P49 |  |  | 29563 | 0.000 | 0.4950 | No |
| 56 | ENSG00000234379 | HMGB1P48 |  |  | 33347 | NaN | 0.4311 | No |
| 57 | ENSG00000260818 | UNGP1 |  |  | 35952 | NaN | 0.3871 | No |
| 58 | ENSG00000213467 | HMGB1P37 |  |  | 36029 | NaN | 0.3859 | No |
| 59 | ENSG00000215873 | FEN1P1 |  |  | 37119 | NaN | 0.3676 | No |
| 60 | ENSG00000271237 | HMGB1P50 |  |  | 37226 | NaN | 0.3659 | No |
| 61 | ENSG00000267444 | SMUG1P1 |  |  | 37927 | NaN | 0.3542 | No |
| 62 | ENSG00000255291 | HMGB1P40 |  |  | 38133 | NaN | 0.3508 | No |
| 63 | ENSG00000248521 | AC092612.1 |  |  | 38333 | NaN | 0.3476 | No |
| 64 | ENSG00000225984 | HMGB1P26 |  |  | 38918 | NaN | 0.3378 | No |
| 65 | ENSG00000251566 | HMGB1P35 |  |  | 39412 | NaN | 0.3296 | No |
| 66 | ENSG00000259699 | HMGB1P8 |  |  | 40782 | NaN | 0.3065 | No |
| 67 | ENSG00000254047 | AC025437.6 |  |  | 40962 | NaN | 0.3036 | No |
| 68 | ENSG00000229316 | HMGB1P45 |  |  | 41161 | NaN | 0.3004 | No |
| 69 | ENSG00000275029 | HMGB1P24 |  |  | 41259 | NaN | 0.2989 | No |
| 70 | ENSG00000237917 | PARP4P1 |  |  | 42034 | NaN | 0.2859 | No |
| 71 | ENSG00000238190 | HMGB1P15 |  |  | 42064 | NaN | 0.2855 | No |
| 72 | ENSG00000230886 | HMGB1P25 |  |  | 42365 | NaN | 0.2805 | No |
| 73 | ENSG00000269583 | L34079.3 |  |  | 43576 | NaN | 0.2602 | No |
| 74 | ENSG00000249338 | HMGB1P47 |  |  | 43898 | NaN | 0.2549 | No |
| 75 | ENSG00000261174 | HMGB1P33 |  |  | 45416 | NaN | 0.2293 | No |
| 76 | ENSG00000244089 | HMGB1P30 |  |  | 45784 | NaN | 0.2232 | No |
| 77 | ENSG00000256514 | AP003419.1 |  |  | 46088 | NaN | 0.2182 | No |
| 78 | ENSG00000258995 | PARP1P2 |  |  | 46263 | NaN | 0.2154 | No |
| 79 | ENSG00000217482 | HMGB1P17 |  |  | 46513 | NaN | 0.2113 | No |
| 80 | ENSG00000213730 | POLD2P1 |  |  | 46754 | NaN | 0.2073 | No |
| 81 | ENSG00000243877 | HMGB1P38 |  |  | 47378 | NaN | 0.1969 | No |
| 82 | ENSG00000227105 | PARP1P1 |  |  | 47523 | NaN | 0.1946 | No |
| 83 | ENSG00000236407 | HMGB1P18 |  |  | 47832 | NaN | 0.1895 | No |
| 84 | ENSG00000203489 | HMGB1P39 |  |  | 50947 | NaN | 0.1369 | No |
| 85 | ENSG00000249065 | PCNAP1 |  |  | 52056 | NaN | 0.1182 | No |
| 86 | ENSG00000254735 | HMGB1P42 |  |  | 52315 | NaN | 0.1140 | No |
| 87 | ENSG00000250721 | HMGB1P22 |  |  | 52617 | NaN | 0.1090 | No |
| 88 | ENSG00000254544 | PCNAP4 |  |  | 52840 | NaN | 0.1054 | No |
| 89 | ENSG00000249745 | HMGB1P28 |  |  | 53451 | NaN | 0.0952 | No |
| 90 | ENSG00000226837 | HMGB1P32 |  |  | 53517 | NaN | 0.0942 | No |
| 91 | ENSG00000214009 | PCNAP3 |  |  | 54078 | NaN | 0.0848 | No |
| 92 | ENSG00000259093 | AL137191.1 |  |  | 54887 | NaN | 0.0713 | No |
| 93 | ENSG00000249263 | PARP4P3 |  |  | 55225 | NaN | 0.0657 | No |
| 94 | ENSG00000248956 | HMGB1P44 |  |  | 55501 | NaN | 0.0611 | No |
| 95 | ENSG00000214821 | HMGB1P4 |  |  | 55945 | NaN | 0.0538 | No |
| 96 | ENSG00000268361 | L34079.1 |  |  | 57590 | NaN | 0.0260 | No |
| 97 | ENSG00000226955 | AC104306.2 |  |  | 58542 | NaN | 0.0100 | No |
Table: GSEA details [plain text format]

  

Fig 2: BASE\_EXCISION\_REPAIR(HSA03410)      
 Blue-Pink O' Gram in the Space of the Analyzed GeneSet

  

Fig 3: BASE\_EXCISION\_REPAIR(HSA03410): Random ES distribution      
 Gene set null distribution of ES for **BASE\_EXCISION\_REPAIR(HSA03410)**
